# Conduction system pacing after atrioventricular junction ablation

**DOI:** 10.1093/europace/euaf108

**Published:** 2025-05-26

**Authors:** Francisco Leyva, Niraj Varma

**Affiliations:** Aston Medical School, Aston University, Aston Triangle, Birmingham B4 7ET, UK; Department of Cardiology, Cleveland Clinic, 9500 Euclid Ave, Cleveland, OH 44106, USA

**This editorial refers to ‘A comparative analysis of conduction system pacing and biventricular pacing in patients undergoing atrioventricular node ablation: a systematic review and meta-analysis’ by A. Mavilakandy *et al.*, https://doi.org/10.1093/europace/euaf106.**

Conduction system pacing (CSP) is evolving as a promising pacing strategy in patients with bradycardia and in patients with heart failure with reduced ejection fraction (HFrEF) and a wide QRS complex. By providing activation through the native conduction system, CSP promises to avoid forced ventricular desynchronization compared to RV apical pacing for bradycardia indications and also to enable resynchronization in patients with a wide intrinsic QRS in the same way as traditional biventricular cardiac resynchronization therapy (BiV-CRT). Effects may be equivalent to or even superior to traditional pacing modes according to multiple recent reports.

In comparison, there are fewer data to show efficacy (or superiority) of CSP in the context of ventricular pacing post-atrioventricular junction ablation (AVJA) for rate control in atrial fibrillation (AF). The practice of AVJA has waned in recent years in favour of AF ablation, but this is not effective for long standing permanent AF. Patients can be medically rate controlled. However, the Ablate and Pace for Atrial Fibrillation—cardiac resynchronization therapy (APAF-CRT trial) indicated that BiV-CRT in combination with AVJA resulted in a 74% reduction in total mortality compared with pharmacological rate control in patients with AF.^1^ This high level of evidence firmly established BiV-CRT as the ‘gold-standard’ pacing modality following AVJA, against which CSP should be compared.^2,3^

In this issue of *EP Europace*, Mavilakandy *et al*. present a metanalysis comparing CSP and BiV-CRT in patients undergoing AVJA.^4^ The narrative review included 1634 CSP recipients and 369 BiV-CRT recipients from 26 studies, all of which were observational, mostly retrospective. No randomized controlled trials were included. The random-effects metanalyses for LVEF and QRS duration were restricted to 294 CSP and 369 BiV-CRT recipients. The authors found that CSP resulted in a higher LVEF and a narrower QRS duration compared with BiV-CRT.

The authors are congratulated for bringing to the fore the available evidence for CSP in patients undergoing AVJA. Several aspects are noteworthy. Analyses were conducted on the few and heterogenous data that are currently available. The CSP group (*n* = 1634) included far fewer patients treated with the currently practised left bundle branch area pacing (LBBAP) compared to those receiving His bundle pacing (HBP). Notably, only a minority of data existed for patients treated with BiV-CRT (*n* = 369) i.e. the desired comparator group. With respect to LVEF, the difference between CSP and BiV-CRT was 3.36%. The 95% confidence intervals (CI) were wide (−0.75–7.47%), most likely because of the heavy influence of two small observational studies from the same centre. The first of these included 24 patients undergoing HBP^5^ and the second included 50 patients undergoing HBP and LBBAP^6^ at the same centre between 2015 and 2022. It is highly likely that patients in the first study^5^ were included in the second.^6^ If the first study had been excluded from the metanalysis, the findings relating to LVEF would have been neutral. Even if we assume no duplication, heterogeneity was substantial (I^2^ = 68.5%), raising doubts as to whether the findings are generalizable. The largest studies^7,8^ found no effect of CSP on LVEF above BiV-CRT. Either way, it is doubtful whether a LVEF improvement of 3.35% is clinically measurable when the quoted standard deviations for LVEF in most centres are ∼5%.

The authors also stated that CSP resulted in a narrower QRS duration (QRSd), with difference of −35.8 ms. Again, the 95% CI were extremely wide (−61.8 to −9.72 ms) and heterogeneity unacceptably high ((I^2^ = 96.3%), raising concerns about generalizability. As for LVEF, the effect on QRS duration was heavily influenced by two small studies from the same centre.

It is difficult to draw any conclusions regarding the narrative review of other measures, as a metanalyses were not formally presented. It is mentioned that CSP showed a ‘non-significant reduction in hospitalization’, but the *P* value was 0.47. The same claim was made for mortality, which was associated with a *P* value of 0.26. Clearly, CSP had no effect on mortality or hospitalization in this analysis.

In conclusion, the authors’ claim, based on limited data from very few patients, that CSP conveys a superior electrocardiographic and echocardiographic response compared to trans-CV CRT is highly questionable. Nor can they claim any favourable effects on mortality or hospitalization, as the analysis clearly show. We should be careful not to overinterpret the findings of metanalyses of observational studies, particularly when the level of heterogeneity is as high as in the present metanalysis. In addition, we should question whether small changes in measures such as LVEF or QRS duration are clinically meaningful, and whether they are proven surrogates of mortality and morbidity. Further data for CSP following AVJA will be become available from ongoing trials (e.g. ABACUS trial; NCT06207383) although none of these compare CSP to BiV-CRT. The definitive question as to whether CSP is superior or non-inferior to BiV-CRT should be addressed in adequately powered randomized, controlled trials using mortality and morbidity endpoints. Until then, the results of APAF-CRT should be considered standard of care and CSP considered as a ‘bail-out’ pacing strategy following AVJA for AF if trans-CV CRT is not technically possible.

## Data Availability

This editorial is based on data published in the related manuscript. No new data has been used in this editorial.
